# Impact of frailty on perioperative outcomes following percutaneous nephrolithotomy in older persons: evidence from the US Nationwide Inpatient Sample

**DOI:** 10.1007/s00240-024-01595-y

**Published:** 2024-06-19

**Authors:** You-Chiuan Chien, Pao-Hwa Chen, Yaw-Jen Chang

**Affiliations:** 1 Department of Mechanical Engineering, Chung Yuan Christian University, Chung Li District, No. 200, Zhongbei Rd., Zhongli Dist, Taoyuan, 320314 Taiwan (R.O.C.); 2 Tai-An Hospital, Taichung, 401007 Taiwan; 3 Department of Surgery, Division of Urology, Changhua Christian Hospital, 135, Nanxiao St, Changhua, Changhua 500209 Taiwan

**Keywords:** Frailty, Kidney stones, Percutaneous nephrolithotomy (PCNL), Perioperative outcomes, Hospital frailty risk score (HFRS), Nationwide inpatient Sample (NIS)

## Abstract

To evaluate the impact of frailty on perioperative outcomes of older patients undergoing PCNL, utilizing the US Nationwide Inpatient Sample (NIS) database. Data of hospitalized patients ≥ 60 years who received PCNL were extracted from the 2010 to 2020 NIS database, and included demographics, clinical, and hospital-related information. Patients were assigned to low (< 5), medium (5–15), and high frailty risk (> 15) groups based on the hospital frailty risk score (HFRS). Associations between frailty risk and perioperative outcomes including total hospital cost were determined using population-weighted linear and logistic regression analyses. Data of 30,829 hospitalized patients were analyzed (mean age 72.5 years; 55% male; 78% white). Multivariable analyses revealed that compared to low frailty risk, increased frailty risk was significantly associated with elevated in-hospital mortality (adjusted odds ratio (aOR) = 10.70, 95% confidence interval (CI): 6.38–18.62), higher incidence of unfavorable discharge (aOR = 5.09, 95% CI: 4.43–5.86), prolonged hospital length of stay (LOS; aOR = 7.67, 95% CI: 6.38–9.22), increased transfusion risk (aOR = 8.05, 95% CI: 6.55–9.90), increased total hospital costs (adjusted Beta = 37.61, 95% CI: 36.39–38.83), and greater risk of complications (aOR = 8.52, 95% CI: 7.69–9.45). Frailty is a significant prognostic indicator of adverse perioperative outcomes in older patients undergoing PCNL, underscoring importance of recognizing and managing frailty in older patients.

**Supplementary Information:**

The online version contains supplementary material available at 10.1007/s00240-024-01595-y.

## Introduction

Kidney stones are increasingly recognized as a major global health issue, adding burden to health care systems, and impacting on individual well-being [1]. The prevalence of kidney stones increases with age, peaking at about 19% in males over 60 years old [2]. Risk factors for developing kidney stones include male sex, hypertension, obesity, diabetes, and personal lifestyle choices such as alcohol consumption and cigarette smoking [3]. Furthermore, kidney stones are recognized as contributing to increased risk of several severe health conditions, including cardiovascular disease (CVD), chronic kidney disease (CKD), and end-stage renal disease (ESRD) [4–6]. Notably, older patients face a higher risk of complications related to kidney stones, and are twice as likely to require hospitalization [7, 8], which indicates there is a need to provide special attention and care to this population.

Percutaneous nephrolithotomy (PCNL) is the first-line intervention for the management of complex kidney stones, and effectively can remove stones through a minimally invasive approach [9]. Compared to shockwave lithotripsy and flexible ureteroscopy, PCNL is associated with a higher success rate and stone-free rate [10–12]. Despite its advantages, this procedure can lead to potential complications such as bleeding, injury to surrounding structures, infection, and positioning-related injuries [13]. However, enhancements in surgeon skill and procedural modifications have been shown to decrease these risks [14]. A notable advancement is tubeless PCNL, which offers several benefits over standard PCNL, including shorter hospital stays, quicker recovery times, and reduced urine leakage [15].

The aging demographic presents a unique challenge, as older adults often exhibit frailty, a multifaceted clinical syndrome characterized by diminished physiological reserves and increased vulnerability to stressors [16]. Recent research has continuously shown that frailty independently predict higher risks of unfavorable outcomes, including increased in-hospital mortality, postoperative complications, and prolonged hospital length of stays (LOS) in multiple surgical settings [17–19]. However, to date, few prior studies have investigated the role of frailty in outcomes of PCNL. Therefore, our study aims to evaluate the impact of frailty on PCNL outcomes of older patients with kidney stones, using a comprehensive national database from the United States (US).

## Methods

### Data source

This population-based retrospective study utilized data from the US Nationwide Inpatient Sample (NIS) database, maintained by the Healthcare Cost and Utilization Project (HCUP) of the US National Institutes of Health (NIH). This database compiles in-depth details on roughly 8 million hospital stays annually, covering patient demographic information, diagnosis and treatment details, admission and discharge times, LOS, and features of the hospitals. Further details on the NIS database can be accessed at: https://hcup-us.ahrq.gov/nisoverview.jsp.

### Ethics statement

Data for the study was sourced from the Online HCUP Central Distributor website (https://www.distributor.hcup-us.ahrq.gov/), adhering to the NIS data-use agreement via HCUP, certified under number HCUP-71JWX29J8. The research, centered on secondary analysis of the NIS database, did not engage patients or the public directly.

### Patient selection

The study included patients ≥ 60 years old who were hospitalized and received PCNL between 2010 and 2020. Patients were identified in the NIS database using International Classification of Diseases (ICD) codes. Exclusion criteria included patients with missing information on age, sex, outcome variables, or dataset sample weights. The ICD codes used in this study are shown in Supplementary Table S1.

### Study outcomes

In-hospital outcomes evaluated were in-hospital mortality, unfavorable discharge, prolonged LOS, transfusion, total hospital costs, and perioperative complications. Unfavorable discharge was defined as discharge to a long-term care facility. Prolonged LOS was defined as ≥ the 75th percentile LOS of the study population. Perioperative complications included acute myocardial infarction (AMI), cerebral vascular accident (CVA), venous thromboembolism (VTE), pneumonia, sepsis, infection, respiratory failure, mechanical ventilation, acute kidney injury (AKI), shock, hemorrhage, wound complication, device complication, nervous system complication, and digestive system complication.

### Hospital frailty risk score (HFRS)

The HFRS was introduced by Gilbert et al. [20] and has been meticulously validated using a comprehensive range of ICD diagnostic codes that signify conditions associated with frailty. The development process involved an extensive study of 1.04 million hospitalized individuals ≥ 75 years old. These codes encompass conditions such as chronic pulmonary disease, heart failure, and volume depletion. To categorize patients based on their frailty risk, we utilized the HFRS, assigning patients into 1 of 3 groups: those with low frailty risk (a score of < 5, also referred to as “non-frail”), medium frailty risk (a score between 5 and 15), and high frailty risk (a score > 15). While the HFRS spans from 0 to 99, these specific cut-offs were established in the original validation study. These thresholds were carefully chosen to most effectively differentiate between individuals in terms of their health outcomes, thereby providing a precise and meaningful assessment of frailty risk. The HFRS has been widely applied in various studies using administrative codes [21, 22].

### Covariates

The data analyzed included patient age, sex, race, income, insurance type, and admission categories. Major comorbidities included coronary artery disease (CAD), congestive heart failure, diabetes, cerebrovascular disease, chronic pulmonary disease, CKD, severe liver disease, rheumatic disease, and any malignancy. The Charlson Comorbidity Index (CCI) was used to evaluate the severity of these comorbidities. We also collected data on hospital features, including bed size and location/teaching status, offering a comprehensive view of patient-related information.

### Statistical analysis

The NIS database includes a 20% sample of US annual inpatient admissions, weighted samples (before 2011 using TRENDWT and after 2012 using DISCWT), stratum (NIS_STRATUM), and cluster (HOSPID) were used to produce national estimates for all analyses. The SURVEY procedure in SAS performs analysis for sample survey data. Patient descriptive statistics were presented as number (n) and weighted percentage (%), or mean and standard error (SE). Categorical data were analyzed by the PROC SURVEYFREQ statement, and continuous data by the PROC SURVEYREG statement. Logistic regressions were performed using the PROC SURVEYLOGISTIC statement to determine the associations between HFRS and in-hospital mortality, unfavorable discharge, prolonged LOS, transfusion, any complications, and results were reported as odds ratio (OR) and 95% confidence interval (CI). Linear regressions were performed using the PROC SURVEYREG statement to determine factors associated with total hospital cost. Multivariable regression was adjusted for variables that were significant (*p* < 0.05) in the univariate analysis (except for CCI). The area under the receiver operating characteristic (ROC) curve (AUC) was used to check the performances of different models for predicting in-hospital mortality. All p-values were 2-sided, and values of *p* < 0.05 were considered statistically significant. All statistical analyses were performed using the statistical software package SAS software version 9.4 (SAS Institute Inc., Cary, NC, USA).

## Results

### Patient selection

**Fig. 1:**
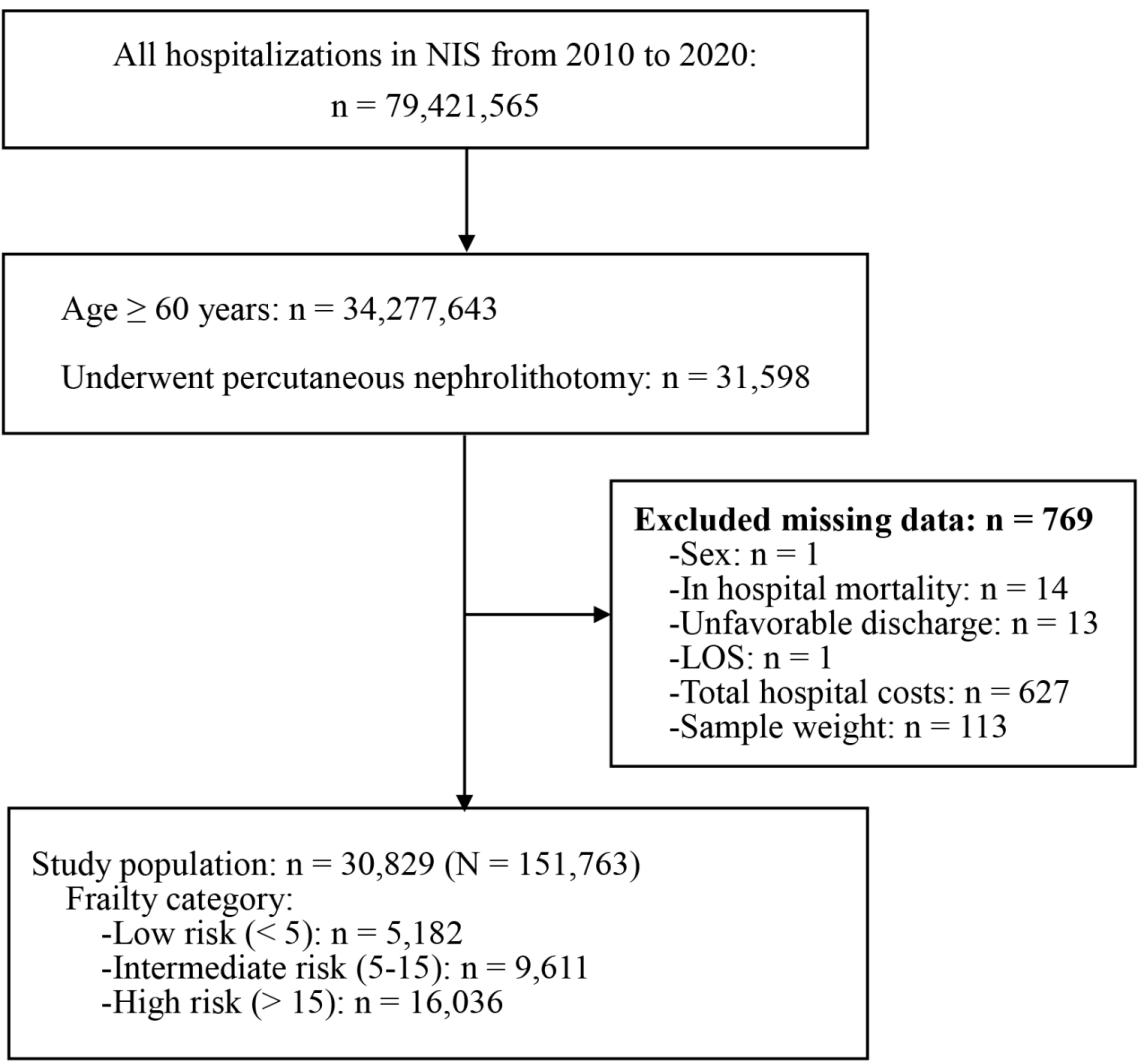
Flow diagram of patient selection

The patient selection process is depicted in Fig. 1. A total of 31,598 patients ≥ 60 years old and received PCNL were identified in the 2010 to 2020 NIS database. Patients with missing information on sex, study outcomes, and sample weight (*n* = 769) were excluded. Finally, 30,829 patients were included in the study (representing 151,763 hospitalized patients in the US after weighting) (Fig. 1).

### Patient characteristics

Patient information including demographic information, comorbidities, outcomes, and hospital characteristics are shown in Table 1. The mean age of the patients was 72.5 years, 54.9% were males, and 78.3% were white. Compared with patients with low and intermediate risk of frailty, those at high risk were older, and had the highest proportion of males, major comorbidities, and higher CCI. Additionally, patients at high risk of frailty had the greatest proportion of insurance coverage by Medicare or Medicaid, were admitted emergently, admitted in urban-teaching hospitals, and in the Western US.

**Table 1:** Characteristics of the study population

	All patients (*N* = 30,829)	HFRS			
		Low risk (< 5)	Intermediate risk (5–15)	High risk (> 15)	*p*-value
		(*n* = 5,182)	(*n* = 9,611)	(*n* = 16,036)	
*Outcomes*					
In hospital mortality	981 (3.2)	15 (0.3)	200 (2.1)	766 (4.8)	**< 0.001**
Unfavorable discharge ^a^	7,277 (24.3)	277 (5.4)	1,569 (16.6)	5,431 (35.5)	**< 0.001**
Prolong LOS ^a, b^	6,993 (23.4)	176 (3.4)	1,478 (15.7)	5,339 (34.9)	**< 0.001**
Total hospital costs, per 1,000 dollars	83.0 ± 0.9	52.9 ± 0.7	70.2 ± 1.1	100.5 ± 1.3	**< 0.001**
Any complications	20,781 (67.4)	1,163 (22.6)	5,837 (60.8)	13,781 (85.9)	**< 0.001**
AMI	749 (2.4)	12 (0.2)	146 (1.5)	591 (3.7)	**< 0.001**
CVA	515 (1.7)	10 (0.2)	109 (1.1)	396 (2.5)	**< 0.001**
VTE	1,618 (5.2)	59 (1.2)	382 (4.0)	1,177 (7.3)	**< 0.001**
Pneumonia	1,426 (4.6)	25 (0.5)	262 (2.7)	1,139 (7.1)	**< 0.001**
Sepsis	10,665 (34.6)	71 (1.4)	1,977 (20.6)	8,617 (53.7)	**< 0.001**
Infection	11,881 (38.5)	201 (3.9)	2,504 (26.1)	9,176 (57.2)	**< 0.001**
Respiratory failure	2,863 (9.3)	38 (0.7)	456 (4.7)	2,369 (14.8)	**< 0.001**
Mechanical ventilation	1,567 (5.1)	19 (0.4)	229 (2.4)	1,319 (8.2)	**< 0.001**
Acute kidney injury	15,015 (48.7)	378 (7.3)	3,838 (39.9)	10,799 (67.3)	**< 0.001**
Shock	2,962 (9.6)	22 (0.4)	445 (4.6)	2,495 (15.5)	**< 0.001**
Hemorrhage	37 (0.12)	2 (0.04)	6 (0.06)	29 (0.18)	**0.006**
Wound complication	711 (2.3)	33 (0.6)	192 (2.0)	486 (3.0)	**< 0.001**
Device complication	542 (1.7)	52 (1.0)	142 (1.5)	348 (2.2)	**< 0.001**
Nervous system	1,109 (3.7)	498 (9.7)	542 (5.7)	69 (0.4)	**< 0.001**
Digestive system	816 (2.6)	84 (1.6)	254 (2.7)	478 (3.0)	**< 0.001**
Transfusion	5,309 (17.1)	121 (2.3)	1,204 (12.5)	3,984 (24.8)	**< 0.001**
*Demography*					
Age, years	72.5 ± 0.1	69.6 ± 0.1	71.9 ± 0.1	73.9 ± 0.1	**< 0.001**
60–69	13,268 (43.1)	2,974 (57.4)	4,387 (45.7)	5,907 (36.9)	**< 0.001**
70–79	10,291 (33.4)	1,618 (31.2)	3,241 (33.7)	5,432 (33.9)	
80+	7,270 (23.5)	590 (11.3)	1,983 (20.6)	4,697 (29.2)	
*Sex*					**0.022**
Male	16,910 (54.9)	2,755 (53.1)	5,293 (55.1)	8,862 (55.3)	
Female	13,919 (45.1)	2,427 (46.9)	4,318 (44.9)	7,174 (44.7)	
*Race*					**< 0.001**
White	22,658 (78.3)	3,909 (79.9)	7,086 (79.0)	11,663 (77.3)	
Black	2,767 (9.6)	346 (7.1)	817 (9.1)	1,604 (10.7)	
Hispanic	1,919 (6.6)	319 (6.6)	591 (6.6)	1,009 (6.7)	
Other	1,599 (5.5)	314 (6.4)	477 (5.3)	808 (5.4)	
Missing	1,886	294	640	952	
*Income*					0.302
Q1	7,107 (23.6)	1,151 (22.7)	2,225 (23.7)	3,731 (23.8)	
Q2	7,407 (24.5)	1,269 (25.0)	2,336 (24.8)	3,802 (24.2)	
Q3	7,966 (26.3)	1,302 (25.6)	2,497 (26.4)	4,167 (26.5)	
Q4	7,745 (25.6)	1,361 (26.7)	2,374 (25.1)	4,010 (25.5)	
Missing	604	99	179	326	
*Insurance status / Primary Payer*					**< 0.001**
Medicare / Medicaid	24,108 (78.3)	3,564 (68.9)	7,383 (76.9)	13,161 (82.1)	
Private including HMO	5,511 (17.9)	1,393 (26.9)	1,835 (19.1)	2,283 (14.3)	
Self-pay/ No charge / Other	1,170 (3.8)	219 (4.2)	376 (3.9)	575 (3.6)	
Missing	40	6	17	17	
*Admission type*					**< 0.001**
Elective	11,290 (36.8)	3,783 (73.6)	4,136 (43.2)	3,371 (21.1)	
Emergent	19,446 (63.2)	1,367 (26.4)	5,454 (56.8)	12,625 (78.9)	
Missing	93	32	21	40	
*Major comorbidities*					
Coronary artery disease	6,728 (21.8)	680 (13.1)	1,862 (19.3)	4,186 (26.1)	**< 0.001**
Congestive heart failure	3,516 (11.4)	178 (3.5)	842 (8.8)	2,496 (15.6)	**< 0.001**
Diabetes	9,062 (29.4)	1,342 (26.0)	2,613 (27.2)	5,107 (31.9)	**< 0.001**
Cerebrovascular disease	1,360 (4.4)	23 (0.4)	210 (2.2)	1,127 (7.0)	**< 0.001**
Chronic pulmonary disease	5,283 (17.2)	612 (11.8)	1,505 (15.7)	3,166 (19.8)	**< 0.001**
Chronic kidney disease	9,400 (30.5)	394 (7.7)	2,253 (23.5)	6,753 (42.1)	**< 0.001**
Severe liver disease	144 (0.5)	5 (0.1)	34 (0.4)	105 (0.7)	**< 0.001**
Rheumatic disease	630 (2.0)	79 (1.5)	192 (2.0)	359 (2.2)	**0.006**
Any malignancy	10,458 (33.9)	1,023 (19.7)	3,364 (34.9)	6,071 (37.8)	**< 0.001**
*Charlson comorbidity index*					**< 0.001**
0–2	20,547 (66.7)	4,430 (85.4)	6,594 (68.7)	9,523 (59.4)	
3–5	4,837 (15.7)	384 (7.5)	1,315 (13.7)	3,138 (19.6)	
6+	5,445 (17.6)	368 (7.1)	1,702 (17.7)	3,375 (21.0)	
*Hospital bed size*					0.455
Small	3,252 (10.5)	564 (10.9)	998 (10.3)	1,690 (10.5)	
Medium	7,313 (23.9)	1,234 (24.0)	2,223 (23.3)	3,856 (24.2)	
Large	20,162 (65.6)	3,367 (65.1)	6,347 (66.4)	10,448 (65.3)	
Missing	102	17	43	42	
*Location / Teaching status*					**< 0.001**
Rural	1,034 (3.3)	187 (3.6)	362 (3.7)	485 (3.0)	
Urban nonteaching	8,870 (28.7)	1,205 (23.2)	2,630 (27.4)	5,035 (31.3)	
Urban teaching	20,823 (68.0)	3,773 (73.2)	6,576 (68.9)	10,474 (65.7)	
Missing	102	17	43	42	
*Hospital region*					**< 0.001**
Northeast	7,737 (25.0)	1,350 (25.8)	2,551 (26.5)	3,836 (23.9)	
Midwest	7,138 (23.0)	1,181 (22.7)	2,234 (23.1)	3,723 (23.1)	
South	10,238 (33.4)	1,759 (34.1)	3,210 (33.6)	5,269 (33.0)	
West	5,716 (18.6)	892 (17.3)	1,616 (16.9)	3,208 (20.0)	

*Abbreviations* HFRS, hospital frailty risk score; LOS, length of hospital stay; AMI, acute myocardial infarction; CVA, cerebral vascular accident; VTE, venous thromboembolism; HMO, Health Maintenance Organization

Continuous variables are presented as mean ± SE; categorical variables are presented as unweighted counts (weighted percentage)

p-values < 0.05 are shown in bold

^a^ Excluding patients who died in the hospital

^b^ Length of hospital stay > 75th percentile: 10 days

Compared to the low and intermediate frailty risk groups, the high frailty risk group had a significantly higher percentage of in-hospital mortality (4.8% vs. 0.3–2.1%, *p* < 0.001), unfavorable discharge (35.5% vs. 5.4–16.6%, *p* < 0.001), prolonged LOS (34.9% vs. 3.4–15.7%, *p* < 0.001), transfusion (24.8% vs. 2.3–12.5%, *p* < 0.001), and overall complications (85.9% vs. 22.6–60.8%, *p* < 0.001). In addition, the high frailty risk group had significantly higher total hospital costs (100,500 USD vs. 52,900 to 70,200 USD, *p* < 0.001) than the other groups (Table 1).

### Associations between in-hospital outcomes and HFRS-defined frailty

**Fig. 2:**
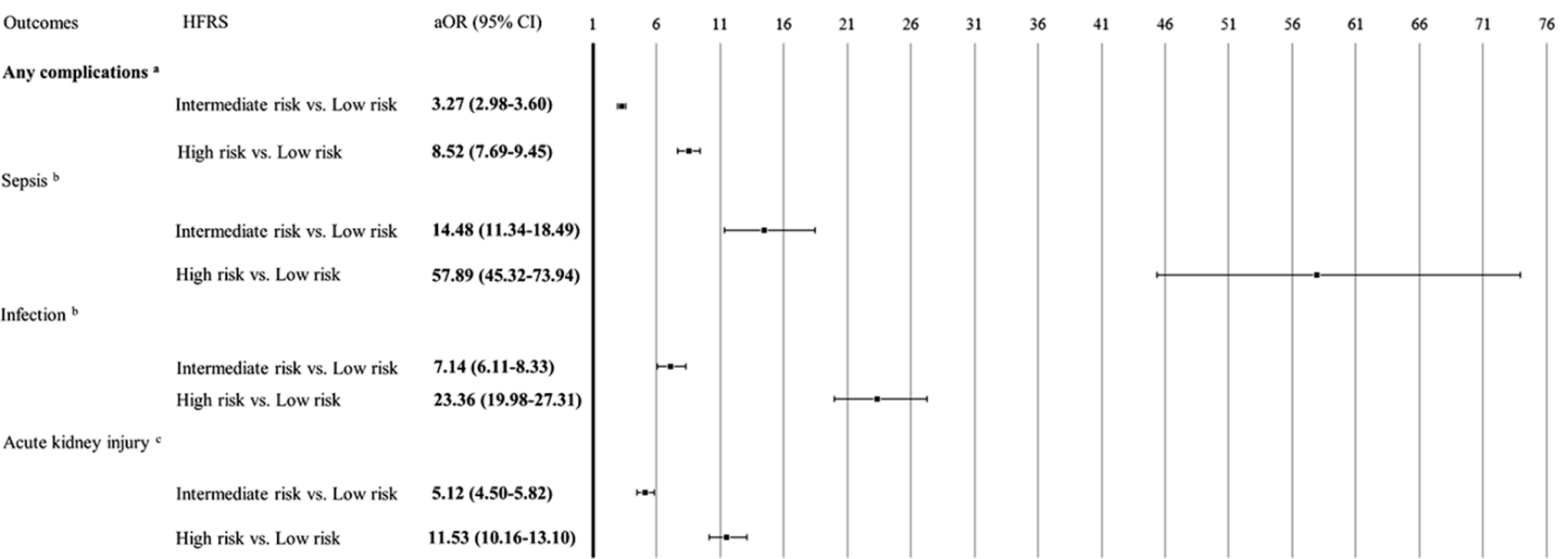
Associations between complications and HFRS. *Abbreviations* CCI, Charlson comorbidity index; HFRS, hospital frailty risk score; aOR, adjusted odds ratio; CI, confidence interval. *p*-values < 0.05 are shown in bold. ^a^ Adjusted for variables that were significant (*p* < 0.05) in the univariate analysis (except for CCI), including age (continuous), sex, race, insurance status / primary payer, admission type, coronary artery disease, congestive heart failure, diabetes, cerebrovascular disease, chronic pulmonary disease, chronic kidney disease, severe liver disease, any malignancy, location/teaching status, and hospital region. ^b^ Adjusted for variables that were significant (*p* < 0.05) in the univariate analysis (except for CCI), including age (continuous), sex, race, insurance status / primary payer, admission type, coronary artery disease, congestive heart failure, diabetes, cerebrovascular disease, chronic pulmonary disease, chronic kidney disease, severe liver disease, rheumatic disease, any malignancy, location/teaching status, and hospital region. ^c^ Adjusted for variables that were significant (*p* < 0.05) in the univariate analysis (except for CCI), including age (continuous), sex, race, insurance status / primary payer, admission type, coronary artery disease, congestive heart failure, cerebrovascular disease, chronic pulmonary disease, chronic kidney disease, rheumatic disease, severe liver disease, any malignancy, location/teaching status, and hospital region

The associations between outcomes and HFRS-defined frailty are summarized in Tables 2 and 3, and Fig. 2. After adjustment in the multivariable analysis, we found that the intermediate and high frailty risk groups had a significantly increased risk for in-hospital mortality (adjusted odds ratio [aOR] = 5.52, 95% confidence interval [CI]: 3.14–9.70, *p* < 0.001; aOR = 10.70, 95% CI: 6.38–18.62, *p* < 0.001, respectively), unfavorable discharge (aOR = 2.45, 95% CI: 2.13–2.82, *p* < 0.001; aOR = 5.09, 95% CI: 4.43–5.86, *p* < 0.001, respectively), prolonged LOS (aOR = 3.33, 95% CI: 2.77–3.99, *p* < 0.001; aOR = 7.67, 95% CI: 6.38–9.22, *p* < 0.001, respectively), and transfusion (aOR = 4.04, 95% CI: 3.29–4.95, *p* < 0.001; aOR = 8.05, 95% CI: 6.55–9.90, *p* < 0.001, respectively) compared to the low risk group (Table 2).

**Table 2:** Associations between in-hospital mortality, unfavorable discharge, prolonged LOS, and transfusion and HFRS

Outcomes	HFRS								
	Intermediate risk vs. Low risk				High risk vs. Low risk				
	OR (95% CI)	*p*-value	aOR (95% CI)	*p*-value	OR (95% CI)	*p*-value	aOR (95% CI)	*p*-value	
In hospital mortality ^b^	7.34 (4.41–12.20)	**< 0.001**	5.52 (3.14–9.70)	**< 0.001**	17.31 (10.56–28.39)	**< 0.001**	10.70 (6.15–18.62)	**< 0.001**	
Unfavorable discharge ^a, c^	3.52 (3.09–4.02)	**< 0.001**	2.45 (2.13–2.82)	**< 0.001**	9.72 (8.55–11.04)	**< 0.001**	5.09 (4.43–5.86)	**< 0.001**	
Prolonged LOS ^a, d^	5.28 (4.44–6.28)	**< 0.001**	3.33 (2.77–3.99)	**< 0.001**	15.24 (12.80-18.15)	**< 0.001**	7.67 (6.38–9.22)	**< 0.001**	
Transfusion ^e^	5.96 (4.91–7.23)	**< 0.001**	4.04 (3.29–4.95)	**< 0.001**	13.78 (11.35–16.73)	**< 0.001**	8.05 (6.55–9.90)	**< 0.001**	

*Abbreviations* CCI, Charlson comorbidity index; HFRS, hospital frailty risk score; LOS, length of hospital stay; OR, odds ratio; aOR, adjusted odds ratio; CI, confidence interval

*p*-values < 0.05 are shown in bold

^a^ Excluding patients who died in the hospital

^b^ Adjusted for variables that were significant (*p* < 0.05) in the univariate analysis (except for CCI), including age (continuous), race, insurance status / primary payer, admission type, coronary artery disease, congestive heart failure, diabetes, cerebrovascular disease, chronic pulmonary disease, chronic kidney disease, severe liver disease, any malignancy, location/teaching status, and hospital region

^c^ Adjusted for variables that were significant (*p* < 0.05) in the univariate analysis (except for CCI), including age (continuous), sex, race, insurance status / primary payer, admission type, coronary artery disease, congestive heart failure, diabetes, cerebrovascular disease, chronic pulmonary disease, chronic kidney disease, severe liver disease, rheumatic disease, any malignancy, hospital bed size, location/teaching status, and hospital region

^d^ Adjusted for significant variables (*p* < 0.05) in the univariate analysis (except for CCI), including age (continuous), race, income, insurance status / primary payer, admission type, coronary artery disease, congestive heart failure, cerebrovascular disease, chronic pulmonary disease, chronic kidney disease, severe liver disease, any malignancy, hospital bed size, location/teaching status, and hospital region

^e^ Adjusted for significant variables (*p* < 0.05) in the univariate analysis (except for CCI), including age (continuous), race, income, insurance status / primary payer, admission type, coronary artery disease, congestive heart failure, cerebrovascular disease, chronic kidney disease, severe liver disease, any malignancy, location/teaching status, and hospital region

^f^ Adjusted for significant variables (*p* < 0.05) in the univariate analysis (except for CCI), including age (continuous), sex, race, insurance status / primary payer, admission type, coronary artery disease, congestive heart failure, diabetes, cerebrovascular disease, chronic pulmonary disease, chronic kidney disease, severe liver disease, any malignancy, location/teaching status, and hospital region

**Table 3:** Associations between total hospital costs and HFRS

Outcome	HFRS								
	Intermediate risk vs. Low risk				High risk vs. Low risk				
	Beta (95% CI)	*p*-value	aBeta (95% CI)	*p*-value	Beta (95% CI)	*p*-value	aBeta (95% CI)	*p*-value	
Total hospital costs ^a, b^	17.27 (14.96–19.58)	**< 0.001**	12.34 (11.18–13.50)	**< 0.001**	47.59 (44.91–50.27)	**< 0.001**	37.61 (36.39–38.83)	**< 0.001**	

*Abbreviations* CCI, Charlson comorbidity index; HFRS, hospital frailty risk score; aBeta, adjusted Beta; CI, confidence interval

*p*-values < 0.05 are shown in bold

^a^ Per 1,000 US dollars

^b^ Adjusted for significant variables (*p* < 0.05) in the univariate analysis (except for CCI), including age (continuous), race, income, insurance status / primary payer, admission type, coronary artery disease, congestive heart failure, diabetes, cerebrovascular disease, chronic pulmonary disease, chronic kidney disease, severe liver disease, any malignancy, hospital bed size, location/teaching status, and hospital region

In addition, compared with the low risk group, the intermediate and high frailty risk groups had significantly greater total hospital costs (aBeta = 12.34, 95%CI: 11.18–13.50, *p* < 0.001; aBeta = 37.61, 95%CI: 36.39–38.83, *p* < 0.001, respectively) (Table 3).

Furthermore, the intermediate and high frailty risk groups had a significantly higher risk of any complication (aOR = 3.27, 95% CI: 2.98–3.60, *p* < 0.001; aOR = 8.52, 95% CI: 7.69–9.45, *p* < 0.001, respectively). For specific complications, the intermediate and high frailty risk groups had a significantly greater risk for sepsis (aOR = 14.48, 95% CI: 11.34–18.49, *p* < 0.001; aOR = 57.89, 95% CI: 45.32–73.94, *p* < 0.001, respectively), infection (aOR = 7.14, 95% CI: 6.11–8.33, *p* < 0.001; aOR = 23.36, 95% CI: 19.98–27.31, *p* < 0.001, respectively), and AKI (aOR = 5.12, 95% CI: 4.50–5.82, *p* < 0.001; aOR = 11.53, 95% CI: 10.16–13.10, *p* < 0.001, respectively) than the low risk group (Fig. 2).

### ROC curves of HFRS for the prediction of in-hospital mortality

**Fig. 3:**
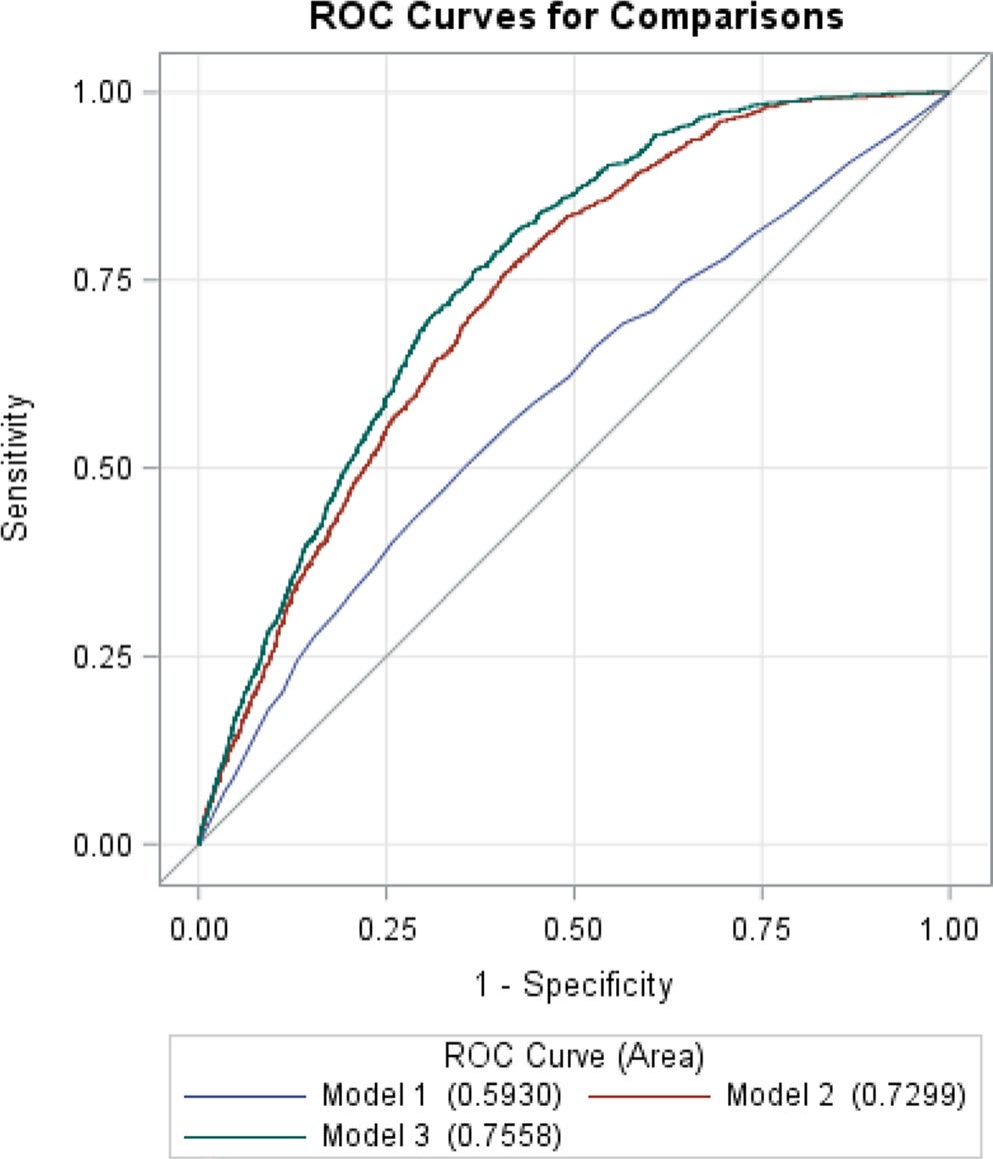
ROC curves of HFRS in the prediction of in-hospital mortality ^a, b, c^*Abbreviations* CCI, Charlson comorbidity index; HFRS, hospital frailty risk score; ROC, receiver operating characteristic. ^a^ Model 1: Age (continuous). ^b^ Model 2: Significant variables (*p* < 0.05) in the univariate analysis (except for CCI), including age (continuous), race, insurance status / primary payer, admission type, coronary artery disease, congestive heart failure, diabetes, cerebrovascular disease, chronic pulmonary disease, chronic kidney disease, severe liver disease, any malignancy, location/teaching status, and hospital region. ^c^ Model 3: Significant variables in the univariate analysis plus HFRS

Figure 3 depicts the performance of the HFRS for predicting in-hospital mortality. Model 1: age (continuous); Model 2: a combination of significant variables (*p* < 0.05) in the univariate analysis (except for CCI); and Model 3: a combination of significant variables in the univariate analysis (except for CCI) plus HFRS. The results showed that Model 3, that included the HFRS, had superior performance (AUC = 0.756, 95% CI: 0.743–0.769) over that of Model 1 (AUC = 0.593, 95% CI: 0.574–0.612) and Model 2 (AUC = 0.730, 95% CI: 0.716–0.744) (Fig. 3).

## Discussion

Our study is the first in the literature to assess the association and predictive ability of frailty on in-patient outcomes of PCNL among older persons. The results indicate a significant and strong correlation between frailty and increased unfavorable perioperative outcomes. Specifically, as identified by the HFRS the high frailty risk group showed increased risks of adverse outcomes, including in-hospital mortality, unfavorable discharge, extended LOS, and the need for transfusions, as compared to the low frailty risk group. Additionally, there was a marked increase in complications such as sepsis, infection, and AKI in patients with high frailty risk. The findings from the ROC analysis indicate that incorporating the HFRS alongside demographic and established clinical risk factors significantly enhanced the predictive accuracy for in-hospital mortality. These findings highlight the importance of considering frailty in the preoperative assessment of older patients undergoing PCNL in order to improve their perioperative outcomes.

With respect to patients with nephrolithiasis, in addition to PCNL, other methods to treat large kidney stones include extracorporeal shockwave lithotripsy (ESWL) and ureteroscopy (URS) [23]. The 3 methods have similar success rates, although surgeon experience and patient selection can play a role in outcomes. Some studies have specifically examined the 3 methods for treating large stones in the geriatric population. Schulz et al. [23] recently performed a literature search to examine the outcomes of the 3 methods in the geriatric population. The authors reported that PCNL had a slightly higher rate of minor complications, but comparable stone free rate and operative time to the other 2 methods. There were minimal complications observed with ESWL and URS, and the clinical success rates were similar between the 2 methods. Liu et al. [24] focused their study on PCNL in elderly patients, with an average age of 75 years, underscoring the particular importance and considerations of this procedure in the geriatric population. The multivariable analysis found that American Society of Anesthesiology (ASA) class III was an independent risk factor for postoperative complications.

Several studies have shown that frailty is predictive of worse outcomes in many different medical fields including general clinical outcomes for hospitalized older adults [16], neurosurgical outcomes in patients with brain tumors [17], older patients with trauma undergoing surgery [18], and older patients undergoing general surgical procedures [19]. Relatively few studies have delved into the impact of preoperative frailty on outcomes in urological conditions, rendering our study novel. It stands out as the first to specifically investigate how frailty affects outcomes of PCNL. Nevertheless, a recent review of the literature by Aro et al. [25] examined the usefulness of frailty assessment in urological stone surgery. Overall, the authors concluded that there is a relation between frailty and outcomes of urological surgeries for stone, and that screening for frailty using an office-based validated tool should be done as part of the preoperative work-up. Another study by Suskind et al. [26] investigated the effect of frailty on the outcomes of 19 different urological procedures, and included about 21,000 cases. The analysis showed that regardless of the type of procedure, preoperative frailty was associated with an increased risk of discharge to a skilled or assisted living facility. A review of the literature by Parikh and Sharma [27] concluded that frailty is a significant prognostic indicator for short-term and long-term outcomes following radical cystectomy. Similarly, a systematic review of the literature by Ornaghi et al. [28] reported that preoperative frailty was significantly predictive of higher rates of major complications and postoperative mortality in patients undergoing radical cystectomy. Taylo et al. [29] examined the impact of frailty on health care resource utilization after urologic oncology surgery. The results showed that for each increase in a frailty score, there was an increase in the likelihood of complications, prolonged LOS, discharge to a continued care facility, and readmission within 30 days. Our results largely align with the findings reported in these studies.

Sepsis represents a critical concern in perioperative care. The most notable risk linked to frailty in our observations is the elevated risk of sepsis. This increased susceptibility to sepsis likely serves as the main contributor to the higher rates of in-hospital mortality we observed. Studies have reported an association between frailty and postoperative infection/sepsis in a wilder population beyond PCNL. For example, a study that used the Spanish Minimum Basic Data Set and analyzed the data of about 250,000 patients ≥ 76 years of age who underwent various surgical procedures reported that patients identified as frail by the HFRS had a significantly higher risk of postoperative sepsis than non-frail patients [30]. Furthermore, a previous study reported that, in patients who develop sepsis, preexisting frailty was predictive of worse outcomes, including in-hospital mortality and discharge to a location other than home [31].

Our analysis also indicates that frailty is a significant predictor of transfusion necessity. While previous research notes that blood transfusion rates for percutaneous nephrostomy lie between 1.5% and 3.2%, these rates increase to between 3.8% and 25% for percutaneous nephrolithotomy (PCNL), attributed to the use of larger tracts and more intensive renal manipulation, highlighting the serious risk of hemorrhage [32]. Nonetheless, studies specifically exploring how frailty affects transfusion risk are limited.

### Strengths and limitations

This study boasts several strengths, including its use of a large, nationally representative sample (NIS database), providing a broad and generalizable insight into the impact of frailty on older patients undergoing PCNL across the US. The dataset, encompassing a decade from 2010 to 2020, provides a thorough and up-to-date glimpse into real-world conditions and practices. The robustness of the study is further enhanced by the comprehensive assessment of frailty using the validated HFRS, and a detailed statistical analysis that includes multiple perioperative outcomes, increasing the reliability of the findings. Notably, compared to other methods of assessing frailty, the HFRS has been shown in other studies to have the highest predictive value for adverse surgical outcomes [33].

However, the study is not without limitations. Its retrospective design is prone to inherent biases, such as selection bias and the inability to establish causality. The reliance on ICD codes for identifying patients and determining complications could lead to misclassification errors. Furthermore, the absence of detailed intraoperative data, prescribed medications, laboratory parameters, information on surgeon experience, and post-discharge follow-up outcomes constrains our ability to fully understand the nuanced clinical implications of frailty on PCNL outcomes. Lastly, focusing solely on patients ≥ 60 years old means the findings might not be directly applicable to younger populations who may also present with varying degrees of frailty.

## Conclusion

Our study demonstrates that HFRS-defined frailty is a strong prognostic indicator for adverse short-term outcomes in older patients undergoing PCNL, including increased in-hospital mortality, unfavorable discharge, prolonged LOS, higher total hospital costs, and postoperative complications. This emphasizes the need for careful frailty assessment and the development of strategies for optimizing the management of older persons undergoing PCNL.

## Electronic supplementary material

Below is the link to the electronic supplementary material.

Supplementary Material 1

## Data Availability

The datasets analysed during the current study are available from the corresponding author on reasonable request.
