# Supplementary material for: Impact of frailty on perioperative outcomes following percutaneous nephrolithotomy in older persons: evidence from the US Nationwide Inpatient Sample
Source: Urolithiasis. 2024 Jun 19;52(1):95. doi: 10.1007/s00240-024-01595-y (PMC11186895; doi:10.1007/s00240-024-01595-y)
Supplement: Supplementary file 1 — Supplementary Material 1 [file 240_2024_1595_MOESM1_ESM.docx]

**Supplementary Table 1: ICD codes used in the study.**

|  | ICD 9 Code | ICD 10 Code |
| --- | --- | --- |
| PCNL | **PCS**: 55.03, 55.04, 55.21 | **PCS**: 0T9040Z, 0T9140Z, 0TC03ZZ, 0TC04ZZ, 0TC13ZZ, 0TC14ZZ, 0TF33ZZ, 0TF34ZZ, 0TF43ZZ, 0TF44ZZ, 0TJ54ZZ |
| Transfusion | **PCS**: 99.02, 99.04 | **PCS**: 30233H0, 30233N0, 30233W0, 30243H0, 30243N0, 30243W0 |
| Acute myocardial infarction | **CM**: 410 | **CM**: I21 |
| Cerebrovascular accident | **CM**: 433.01, 433.10, 433.11, 433.21, 433.31, 433.81, 433.91, 434.00, 434.01, 434.11, 434.91, 436, 430, 431 | **CM**: I60, I61, I63, I69 |
| Venous thromboembolism | **CM**: 415, 451-453, 671, 673, 997.2 | **CM**: I260, I269, I801-803, I808, I809, O082, O223, O871, O882, I81, I82 |
| Pneumonia | **CM**: 486, 481, 482.8, 482.3 | **CM**: A48.1, J12 - J18 |
| Sepsis | **CM**: 995.9, 996.64, 038, 999.3, 790.7, 041.x, 785.52 | **CM**: R78.81, A41, R65.2, A42.7, A22.7, B37.7, A26.7, A28.2, A54.86, A32.7, A24.1, A39.2, A20.7, A21.7, A48.3 |
| Infection | **CM**: 001-139, V09.0-V09.6, V09.8-V09.9, 695.81, 998.5, 998.59 | **CM**: L00-L08, A00-B99, T81.43, O86.03, Z16 |
| Respiratory failure | **CM**: 518.5, 518.81-518.84 | **CM**: J95.2-J95.8, J96.00, J96.90, J80, J81.0 |
| Mechanical ventilation | **PCS**: 96.7 | **PCS**: 5A1935Z, 5A1945Z, 5A1955Z |
| Acute kidney injury | **CM**: 584.x | **CM**: N17 |
| Shock | **CM**: 998.0, 785.5, 995.4 | **CM**: R57, T81.1, T88.2, R65.21 |
| Hemorrhage | **CM**: 459.0 | **CM**: R58 |
| Wound complication | **CM**: 998.3, 998.83, 998.0, 998.31, 998.32, 998.33, 998.12-998.13, 998.5 | **CM**: T81.3, K91.7 |
| Device complication | **CM**: 996.1, 996.62, 996.74, 998.4, 998.7, 998.2 | **CM**: T81.5, T81.6 |
| Nervous system | **CM**: 997.0x | **CM**: G00-G99, M79.2 |
| Digestive system | **CM**: 997.4, 998.59, 567.22 | **CM**: K66.0, K65.1, K68.11, K91.8 |
| Coronary artery disease | **CM**: 410-414 | **CM**: I25 |
| Congestive heart failure |  | **CM**: I09.9, I11.0, I13.0, I13.2, I25.5, I42.0, I42.5-I42.9, I43.x, I50.x, P29.0 |
| Diabetes mellitus |  | **CM**: E10-E14 |
| Cerebrovascular disease | **CM**: 362.34, 430-438 | **CM**: G45.x, G46.x, H34.0, I60.x-I69.x |
| Chronic pulmonary disease | **CM**: 416.8, 416.9, 490-505, 506.4, 508.1, 508.8 | **CM**: I27.8, I27.9, J40 -J47, J60-J67,  J68.4, J70.1, J70.3 |
| Chronic kidney disease | **CM**: 403.01, 403.11, 403.91, 404.02, 404.03, 404.12, 404.13, 404.92, 404.93, 582, 583.0-583.7, 585, 586, 588.0, V42.0, V45.1, V56 | **CM**: I12.0, I13.1, N03.2-N03.7, N05.2-N05.7, N18, N19, N25.0, Z49.0-Z49.2, Z94.0, Z99.2 |
| Severe liver disease | **CM**: 456.0-456.2, 572.2-572.8 | **CM**: I85.0, I85.9, I86.4, I98.2, K70.4, K71.1, K72.1, K72.9, K76.5, K76.6, K76.7 |
| Rheumatic disease | **CM**: 446.5, 710.0-710.4, 714.0-714.2, 714.8, 725.x | **CM**: M05.x, M06.x, M31.5, M32.x-M34.x, M35.1, M35.3, M36.0 |
| Any malignancy | **CM**: 140-172, 174-195.8, 200-208, 238.6, 196-199 | **CM**: C00-C26, C30-C34, C37-C41, C43, C45-C58, C60-C76, C81-C85, C88, C90-C97, C77-C80 |

CM, clinical modification; PCS, procedure codes.
